# Predictors of the Course of Post-Traumatic Stress Disorder After Physical Injury Over Two Years

**DOI:** 10.1192/bjo.2024.186

**Published:** 2024-08-01

**Authors:** Jae-Min Kim

**Affiliations:** Chonnam National University Hospital, Gwangju, Republic of Korea

## Abstract

**Aims:**

The course of post-traumatic stress disorder (PTSD) is complex and remains an area of active investigation, as analyses aimed at identifying predictors of PTSD outcomes often produce variable and inconsistent results. This particular study delves into the progression and patterns of PTSD over a two-year period, focusing on individuals who are in the recovery phase from severe physical injuries. The research aims to understand the different trajectories that PTSD can take and to identify the factors that may influence these pathways, with the goal of enhancing our understanding and treatment of this challenging and multifaceted condition.

**Methods:**

Patients were recruited from a trauma center at a university hospital in South Korea between June 2015 and January 2021. At baseline, 1142 patients underwent evaluations encompassing trauma and PTSD-related measures, socio-demographic characteristics, pre-trauma characteristics, and peri-trauma assessments. They were subsequently followed up for PTSD using the Clinician-administered PTSD Scale (CAPS) at 3, 6, 12, and 24 months. The analyzed sample consisted of 1014 patients who were followed up at least once after the baseline and 3-month evaluations. Latent class growth analysis was employed to identify distinct trajectory groups, and logistic regression models to ascertain predictors associated with each trajectory.

**Results:**

The study identified five unique trajectories of PTSD progression among the patients: resilient, worsening/recovery, worsening, recovery, and chronic groups. The "worsening/recovery” trajectory, which indicates patients whose symptoms initially worsened but later improved, was predominantly associated with individuals who had experienced previous traumatic events and those who had sustained injuries from traffic accidents. On the other hand, the "worsening” trajectory, where patients' symptoms continuously deteriorated over time, was linked to individuals with higher education levels and elevated depressive symptoms. The "recovery” trajectory, characterized by a gradual improvement in symptoms, was more common in female patients and those with a history of childhood abuse, traffic-related injuries, a dissociative subtype of PTSD, and higher levels of anxiety and depressive symptoms. Lastly, the "chronic” trajectory, where patients experienced persistently severe symptoms, was predicted by the presence of a dissociative subtype of PTSD and heightened anxiety symptoms. These findings illustrate the diverse paths PTSD can take and highlight the importance of various factors in influencing these trajectories.

**Conclusion:**

These findings highlighted the heterogeneity of PTSD symptom development and thus the importance of considering individual characteristics when assessing and addressing PTSD following severe physical injuries.

